# *Anisakis pegreffii* (Nematoda: Anisakidae) products modulate oxidative stress and apoptosis-related biomarkers in human cell lines

**DOI:** 10.1186/s13071-016-1895-5

**Published:** 2016-11-25

**Authors:** Concetta Maria Messina, Federica Pizzo, Andrea Santulli, Ivana Bušelić, Mate Boban, Stjepan Orhanović, Ivona Mladineo

**Affiliations:** 1 Dipartimento di Scienze della Terra e del Mare DiSTeM, Università degli Studi di Palermo, Laboratorio di Biochimica Marina ed Ecotossicologia, Via G. Barlotta 4, 91100 Trapani, Italy; 2 Institute of Oceanography and Fisheries, Laboratory of Aquaculture, POBox 500, 21000 Split, Croatia; 3 University of Split, Faculty of Natural Sciences, Teslina 12, 21000 Split, Croatia

**Keywords:** *Anisakis pegreffii*, Apoptosis, Fibroblast cell lines HS-68, Inflammation, Oxidative stress

## Abstract

**Background:**

In countries with elevated prevalence of zoonotic anisakiasis and high awareness of this parasitosis, a considerable number of cases that associate *Anisakis* sp. (Nematoda, Anisakidae) and different bowel carcinomas have been described. Although neoplasia and embedded larvae were observed sharing the common site affected by chronic inflammation, no association between the nematode and malignancy were directly proved. Similarly, no data are available about the effect of secretory and excretory products of infecting larvae at the host’s cellular level, except in respect to allergenic interaction.

**Methods:**

To test the mechanisms by which human non-immune cells respond to the larvae, we exposed the fibroblast cell line HS-68 to two *Anisakis* products (ES, excretory/secretory products; and EC, crude extract) and evaluated molecular markers related to stress response, oxidative stress, inflammation and apoptosis, such as p53, HSP70, TNF-α, c-jun and c-fos, employing cell viability assay, spectrophotometry, immunoblotting and qPCR.

**Results:**

Both *Anisakis* products led to increased production of reactive oxygen species (ROS), especially in EC-treated cells. While the ES treatment induces activation of kinases suggesting inflammation and cell proliferation (or inhibition of apoptosis), in EC-treated cells, other signaling pathways indicate the inhibition of apoptosis, marked by strong upregulation of Hsp70. Elevated induction of p53 in fibroblasts treated by both *Anisakis* products, suggests a significantly negative effect on the host DNA.

**Conclusions:**

This study shows that in vitro cell response to *Anisakis* products can result in at least two different scenarios, which in both cases lead to inflammation and DNA damage. Although these preliminary results are far from proving a relationship between the parasite and cancer, they are the first to support the existence of conditions where such changes are feasible.

**Electronic supplementary material:**

The online version of this article (doi:10.1186/s13071-016-1895-5) contains supplementary material, which is available to authorized users.

## Background

Nematodes of genus *Anisakis* exhibit an indirect life-cycle where life-stages are propagated through trophic webs of their marine hosts. Hundreds of fish species act as paratenic hosts and more than thirty cetacean and pinnipeds are recognised as definitive hosts [1]. In some Mediterranean fish species, infection levels can reach up to 300 larvae [2]. Raw or inadequately thermally processed fishery products that are contaminated by third-stage *Anisakis* larvae (L3), represent a risk factor for men, which are accidental hosts for this nematode. This zoonotic disease is termed anisakidosis, when caused by any species of the family Anisakidae or anisakiasis, when caused by any species of the genus *Anisakis*. Penetration of larvae through the gastrointestinal tract induces severe symptoms, although larvae fail to complete their life-cycle in humans (reviewed by [3–5]). Anisakiasis exists in different types; gastric, intestinal and ectopic [6], while gastroallergic anisakiasis is a form of gastric anisakiasis associated with allergic symptoms (e.g. from urticaria to anaphylactic shock) in *Anisakis*-hypersensitized patients [7, 8].

Additionally, cases that associate *Anisakis* sp. and different bowel carcinomas have been described mainly in countries where both the prevalence of anisakiasis and the awareness of this zoonosis is high [9–13]. It is still unclear if two etiologies, e.g. anisakiasis and carcinoma are related or just accidental incidences [14], but it is indicative that in all reported cases, neoplasia and embedded larvae share the common site affected by chronic inflammation. Carcinogenic potential of parasitic Platyhelminthes has been previously recognized and described in opisthorchiasis, caused by the digenean *Opisthorchis viverrini* (Trematoda: Opisthorchidae) endemic in southeast Asia, and schistosomiasis, caused by the digenean *Schistosoma haematobium* (Trematoda: Schistostomatidae). *Opisthorchis* promotes development of bile ducts carcinoma, indirectly caused by mechanical trauma and metabolites secreted during migration. The consequent inflammatory reaction, fibrosis and cell proliferation, epithelia hyperplasia, rodlet cells metaplasia, hyperplastic adenomatosis, all help in building up a susceptibility to DNA damage and mutation. Elevated expression of nitric oxide synthase (NOS) and resulting increase of nitric oxide (NO) as a product of the fulminant host immune response, causes accumulation of endogenous N-nitroso compounds [15–20]. These, along with the exogenous compounds present in the fermented fish products contaminated by *Opisthorchis*, induce DNA alkylation and deamination in infected tissues, and development of carcinoma [21, 22]. In contrast, *Schistosoma* stimulates the onset of bladder carcinoma most likely through mutation in the *KRAS* gene (*Kirsten rat sarcoma* viral oncogene homolog), which is one of the three proto-oncogenes encompassed in *ras* group, inlcuding *HRAS* (*Harvey rat sarcoma* viral oncogene homolog) and *NRAS* (*Neuroblastoma RAS* viral (v-ras) oncogene homologs). Mutations in *KRAS* activate its transcription, consequently stimulating cell proliferation and inhibition of apoptosis, two important mechanisms that regulate the number of cells in the tissues [23].

The effects of *Anisakis* infection or its secretory products have shown contrasting results so far; in some cases, they triggered carcinogenesis, while in others they exhibited cytostatic activity [14, 24, 25]. For example, Th2-associated eosinophilia and IL-4 secretion in *Anisakis* infections have been suggested to decrease tumor growth and initiate antitumor activity. However, Th2 dominance has been also regarded as tumor growth stimulus that promotes angiogenesis and inhibits cell-mediated immunity and tumor cell killing [4]. With the exception of immune cells engaged in allergenic interaction with infective larvae, no data are available on the effect of nematode secretory and excretory products at the non-immune host cellular level, important for evaluation of other pathogenic pathways of the larvae.

In line with this, the aim of our study was to test the effect of two types of *A. pegreffii* products on the human normal fibroblast cell line HS-68; one *A. pegreffii* product was obtained from live and active larvae, excreted and secreted during its tissue penetration in the host (ES), and the other obtained as a crude extract of the whole body of senescent larvae (EC). Subsequently, we assessed the correlation between toxicity, oxidative stress, apoptosis and cancer pathways by evaluating: (i) cellular vitality; (ii) ROS production; and (iii) the expression of molecular markers related to stress response, oxidative stress, inflammation and apoptosis, such as p53, HSP70, TNF-α, c-jun and c-fos.

## Methods

### Parasite isolation and products extraction

Infective third-stage larvae (L3) of *A. pegreffii* were isolated from visceral cavity of the Atlantic horse mackerel (*Trachurus trachurus*) and morphologically identified as *Anisakis* sp. based on mucron and boring tooth presence, and the appearance of the esophagus and ventriculus [26] under a light microscope (Olympus, SZX10). Excretory/secretory (ES) products were obtained as described by [27]; larvae were washed with sterile saline, soaked in 0.1 M glycine solution (pH 2.0) for 30 min, and cultured in RPMI 1640 supplemented with antibiotics in a humid 37 °C and 5% CO_2_ incubator. Daily harvested supernatant of larval culture was filtered and lyophilized until use, when it was resuspended in sterile PBS. To obtain crude extract (EC), nematodes were extensively washed and subsequently homogenized by tissue grinder in cold PBS using a previously described method [28]. Briefly, the homogenate was sonicated, delipidized using n-hexane, centrifuged (8497⋅ *g*/30 min) and supernatant dialyzed overnight in PBS at 4 °C. Protein content of products was measured following [29].

### Cell culture and conditions

Human normal fibroblast cell line HS-68 was obtained from the American Type Culture Collection (ATCC). Cells were maintained at 37 °C and 5% CO_2_, and were grown as a monolayer in flasks in DMEM supplemented with 10% fetal bovine serum (FBS) (Gibco, ThermoFisher Scientific, Waltham, MA, USA).

Both ES and EC obtained in sterile PBS were further diluted in culture media for different treatments. For tests of vitality and ROS production, cells were cultured in 96-well plates, and treated 24 h afterwards. For the assessment of dose-time effect on the vitality, cells were treated 24, 48, 72 and 96 h with ascending doses (from 0.1 to 5%) of both products. In control wells (CO), PBS alone was added to cells at the same volume as products employed in treatments.

For the assessment of protein markers for oxidative, inflammatory and carcinogenic stress, treatments were replicated in Petri dishes in order to obtain adequate quantities of protein and RNA to perform immunoblotting and RT-PCR, respectively. At the end of the experiments, cells were harvested by adding 0.5% trypsine in PBS, centrifuged at 800⋅*g*/10 min and incubated for 30 min on ice in lysis buffer (1% NP-40, 0.5% sodium deoxycholate, 0.1% SDS, cocktail of protease inhibitors) for sonication. Protein concentration was measured according previously described methods [29].

### Cell viability

Cell viability was assayed by MTT (3-(4,5-Dimethylthiazol-2-yl)-2,5-Diphenyltetrazolium Bromide) test according to [30]. After cell exposure to products, 96-well plates were removed from the incubator and to each well 20 μl of MTT solution was added in dim/dark conditions using a covered test tube (for the reagents). The plate was wrapped in aluminum foil and incubated for 2 h at 37 °C. Afterwards, the contents of each well was removed and 200 μl of lysis buffer was added to the wells, including blank wells that served for the calibration of the plate reader. The plate was read at 590 nm using an Opsys MR plate reader (Dynex Technologies inc., Chantilly, VA, USA). For treated cells, viability was expressed as a percentage in respect to the negative controls. Each treatment and each concentration was done in six replicates.

### Evaluation of intracellular ROS

Intracellular ROS were analyzed on cells seeded in 96-well microplates using the dichlorodihydrofluorescein-diacetate (DCF-DA) method, because DCF-DA oxidizes to dichlorodihydrofluorescein (DCF) by ROS [31]. Each well was exposed to 10 μl of DCF-DA in HBSS (5 μg/ml), incubated for 5 min at 37 °C to allow the oxidation of the DCF-DA and successively read on a spectrofluorometer, 485 exc - 530 em (Varian Cary Eclipse; Agilent Technologies, Santa Clara, CA, USA). To further support the presence of ROS, additional wells were treated with the antioxidant N-acetilcysteine that inhibits its formation, according to a standardized protocol [32]. The results were expressed as relative fluorescence/μg of total proteins (rf/μg tp). Each treatment and each concentration was done in six replicates.

### Immunoblotting

Levels of Hsp70, p53 and TNF protein were evaluated by immunoblotting. Equivalent amounts of each protein (20 μg) were separated by SDS-polyacrylamide gel electrophoresis (SDS-PAGE) and transferred to a nitrocellulose membrane using a Trans Blot Turbo Transfer System (Bio-Rad, Hercules, CA, USA). Filters were then used for protein detection by primary antibodies; anti-Hsp70 (rabbit monoclonal anti-Hsp70, Sigma), anti-p53 (mouse monoclonal anti-p53, Sigma), anti-TNF (rabbit polyclonal anti-TNFSF 13B, Sigma, St Louis, MI, USA) and the appropriate anti-mouse or anti-rabbit horseradish peroxidase-conjugated secondary antibody (GAR/M-HRP Bio-Rad). Immuno-reactive signals were detected using enhanced chemo-luminescent (ECL) reagents (Bio-Rad). The correct protein loading was confirmed by red Ponceau staining. Images were obtained, visualized, photographed and digitalized with Chemi Doc XRS (Bio-Rad), and further analyzed with Image Lab software (Bio-Rad) for relative quantification of the bands. The results were expressed as a fold increase of each treatment in relation to the respective control, representing the mean value of three separate experiments.

### Statistical analysis

Data are given as means ± standard deviations. The differences among the mean values were assessed by the Student-Newman-Keuls test. The degree of heterogeneity was assessed by the Cochran test. Differences were considered significant both at *P* < 0.05 and *P* < 0.0001. The relationship between cell viability and ROS production was evaluated by regression analysis and correlation. The analysis was performed using STATISTICA (version 8.0, Statsoft Inc., USA).

### Real-time PCR analysis

Total cellular RNA was prepared using Aurum Total RNA Fatty and Fibrous Tissue Kit (Bio-Rad), and concentrations were assessed spectrophotometrically at 260 nm. The absorbance ratios A260/A280 and A260/A230 were evaluated as indicators of RNA purity. Forty nanograms of RNA were reverse-transcribed for each sample, in a volume of 20 μl by the 5⋅ iScript Reaction Mix Kit (Bio-Rad), according to the manufacturer’s instructions. Target genes for the relative quantification by real-time PCR were *p53*, *c-fos* and *c-jun*, and *GAPDH* and *β-actin* as reference genes (Table 1).

**Table 1:** Primer sequences of target genes used in the study

Gene	Primer (5′–3′)	Annealing temperature (°C)	Accession number
*c-jun*	F: GGATCAAGGCGGAGAGGAA	69	NC_018912.2
	R: TCCAGCCGGGCGATT		
*c-fos*	F: TCACCCGCAGACTCCTTCTC	71.5	NC_018925.2
	R: GTGGGAATGAAGTTGGCACTG		
*p53*	F: AAGAAACCACTGGATGGAGAA	69	NC_018928.2
	R: CAGCTCTCGGAACATCTCGAAA		
*β-actin*	F: AGGCTGTGCTGTCCCTGTAT	70	NC_018928.2
	R: ACCCAAGAAGGAAGGCTGGA		
*GADPH*	F: ACCCACTCCTCCACCTTTGAC	72	NC_018923.2
	R: GTCCACCACCCTGTTGCTGTA		

*Abbreviations*: *F* forward primer, *R* reverse primer

The amplification was performed in a total volume of 20 μl, which contained 0.4 μM of each primer, cDNA diluted 1:10 of the final reaction volume, 1⋅ IQ SYBR Green Supermix (Bio-Rad) and nuclease-free water. Conditions for real-time PCRs were optimized in a gradient cycler (C1000 Touch Thermal Cycler, Bio-Rad) and afterwards the following program was used: an initial activation step at 95 °C for 3 min, followed by 39 cycles of 95 °C for 10 s and 60 °C for 30 s, with a single fluorescence measurement. Melting curve data were collected at 65–95 °C with heating rate of 0.5 °C/cycle and a continuous fluorescence measurement. All reactions were performed three times in duplicate. For each PCR, we checked linear range of a standard curve of 6 serial dilutions. The relative quantification of *p53*, *c-fos*, *c-jun* gene expression was evaluated after normalization with reference genes. Data processing and statistical analysis were performed using CFX Manager Software (Bio-Rad).

## Results

### Cellular vitality

**Fig. 1:**
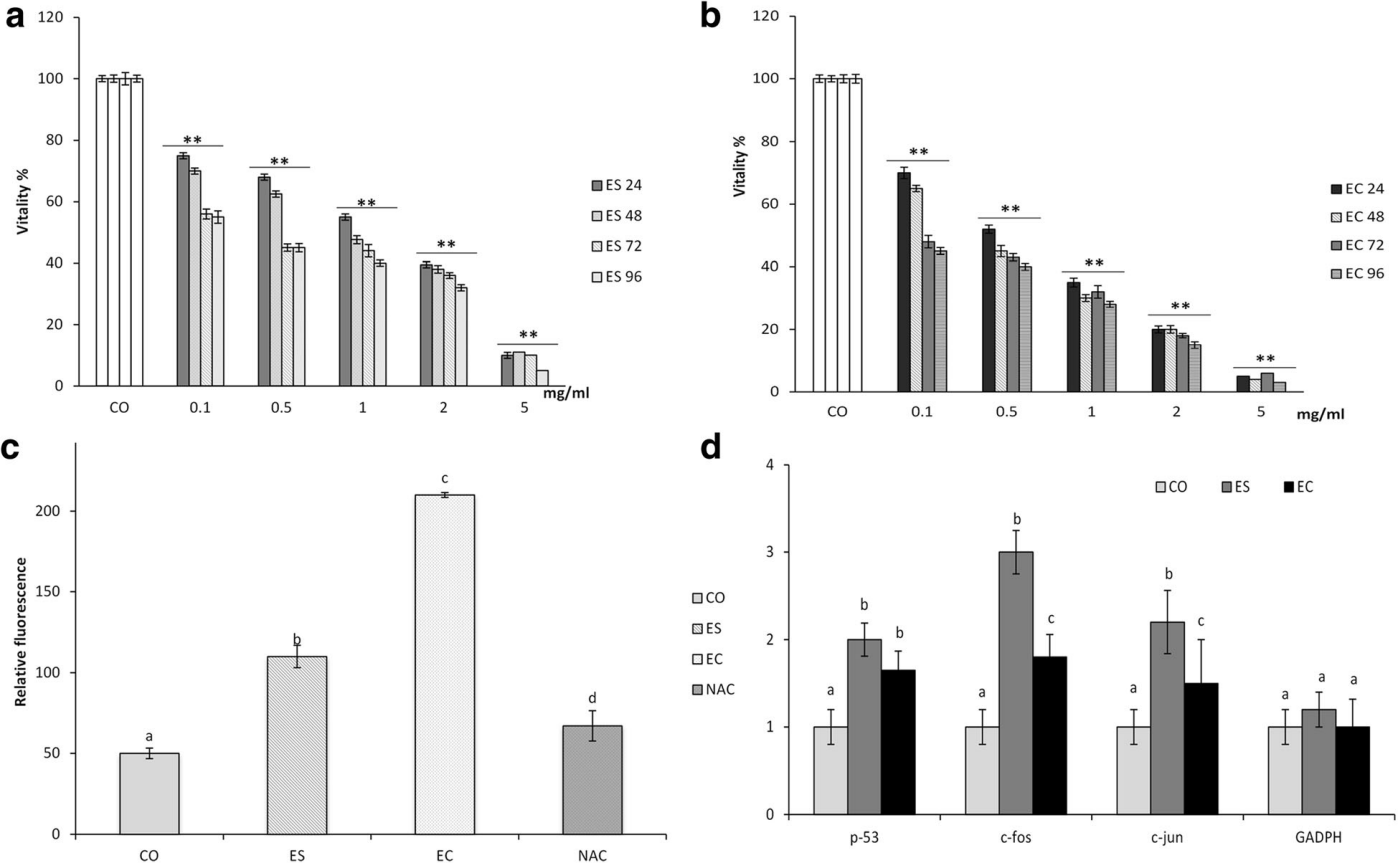
Response of fibroblast HS-68 cells lines to the treatments with *Anisakis* products: **a** Dose-dependent changes in percentage of cell vitality (mean ± SD) after exposure to excretory/secretory (ES) products for 96 h in respect to control cells. ANOVA: ES 24 *F*_(5,30)_ = 2794.7, *P* < 0.0001; ES 48 *F*_(5,30)_ = 2969.5, *P* < 0.0001; ES 72 *F*_(5,30)_ = 1999.3, *P* < 0.0001; ES 96 *F*_(5,30)_ = 2706.6, *P* < 0.0001. **b** Dose-dependent changes in percentage of cell vitality (mean ± SD) after exposure to crude extract products (EC) for 96 h in respect to control cells. ANOVA: EC 24 *F*_(5,30)_ = 3088,8, *P* < 0.0001; EC 48 *F*_(5,30)_ = 3729.97, *P* < 0.0001; EC 72 *F*_(5,30)_ = 4109.5, *P* < 0.0001; EC 96 *F*_(5,30)_ = 3960.1, *P* < 0.0001. **c** Reactive oxygen species (ROS) production, expressed as relative fluorescence/ mg total proteins (means ± SD) in cell after 48 h-exposure to 0.1% concentration of *Anisakis* excretory/secretory (ES) and crude extract (EC) products ANOVA: *F*_(3,20)_ = 857.83, *P* < 0.0001. **d** Fold increase (means ± SD), respect to the control cells, of *p53*, *c-fos*, *c-jun* and *GADPH* mRNA expression after 48 h-exposure to 0.1% concentration of *Anisakis* excretory/secretory (ES) and crude extract (EC) products. ANOVA: p53 *F*_(2,12)_ = 17.4, *P* < 0.0001; c-fos *F*_(2,12)_ = 76.6, *P* < 0.0001; c-jun *F*_(2,12)_ = 54.2, *P* < 0.0001; GADPH *F*_(2,12)_ = 0.95, *P* = 0.415. *Abbreviations*: CO, untreated control cell lines; NAC, synthetic antioxidant N-acetilcysteine

The effect of two products on cell vitality is shown in Fig. 1a, b. Cell progressive reduction in vitality was notable in a dose-dependent manner when both *Anisakis* products were used, in contrast to control treatments (CO) that maintained 100% vitality during the whole experiment.

All employed concentrations of ES induced a highly significant reduction in cell vitality in respect to CO, starting from 0.1% at 24 h (*F*_(5,30)_ = 2794.7, *P* < 0.0001) (Fig. 1a). In general, the same trend was observed in EC-treated cells, in which all treatments after 24 h induced a highly significant vitality reduction in respect to the control (*F*_(5,30)_ = 3088.8, *P* < 0.0001) (Fig. 1b).

The cells were affected by the toxic effect of both treatments in a dose-time dependent manner (Fig. 1a, b): after 96 h, cell viability treated by ES was equal to 5% in respect to CO (*F*_(5,30)_ = 2706.6, *P* < 0.0001), and treated by EC was equal to 3% (*F*_(5,30)_ = 3960.1, *P* < 0.0001).

In order to evaluate the biochemical responses of treated cells, a 0.1% concentration of both products after 48 h was used in all downstream reactions.

### ROS production

When 0.1% concentrations of ES and EC were applied and ROS production measured 48 h post-treatment, statistically significant increase was noted compared to the untreated control (*F*_(3,20)_ = 857.83, *P* < 0.0001), (Fig. 1c). Higher ROS production was observed in EC-treated cells (*P* < 0.0001), while cells pretreated with the antioxidant N-acetilcysteine expressed reduced ROS production, being protected by the compound (*P* < 0.0001). Regression analysis between cell vitality and ROS demonstrated that a relationship exists between the two variables (*r* ^*2*^ = 0.89) (Additional file 1: Figure S1), showing to be also highly correlated.

### Biomolecular markers of oxidative stress, inflammation and apoptosis

An increase in p53 protein (*F*_(2,6)_ = 43.9, *P* < 0.05) and gene expression (*F*_(2,12)_ = 17.4, *P* < 0.0001) levels was observed after 0.1% ES and EC treatments in respect to control (Fig. 1d and Additional file 2: Figure S2). Heat-shock protein 70 levels were significantly increased in EC-treated compared to control cells (*F*_(2,6)_ = 269.4, *P* < 0.0001), as well as ES-treated cells, where expression decreased (Additional file 2: Figure S2). In contrast, TNF protein was significantly increased by ES treatment (*F*_(2,6)_ = 20.6, *P* < 0.05) (Additional file 2: Figure S2). Gene expression analysis of *c-fos* and *c-jun* demonstrated up-regulation in respect to control cells, and significantly higher levels were measured after ES treatment (*F*_(2,12)_ =76.6 *P* < 0.0001; *F*_(2,12)_ = 54.2, *P* < 0.0001) (Fig. 1d).

## Discussion

Two types of *Anisakis* products were used to simulate different scenarios of *Anisakis* infection in men. Experiments were performed on human fibroblast cells, applying concentrations which in vitality tests have proven to have the lowest toxicity and highest cell vitality. In the first scenario, we used excretory and secretory products (ES) of live larvae, mimicking the active penetration of the parasite through the human digestive wall. In vivo, those products encompass a wide array of proteins and glycoproteins, localized primarily in the parasite’s excretory glands, as well as in the ventriculus, intestinal epithelium and basal cuticle layer [33–36]. The largest part of ES products act as potent allergens and immunomodulators during parasite infection, helping larval migration [24, 34, 37], while those with low molecular mass (<10,000 Da) have been reported to have mutagenic properties [38]. While the distinctive feature of anisakid infection are inflammatory lesions supported by eosinophilic infiltration surrounding larval cuticle [39], the invasive capacity of larva is correlated to secretion of its anticoagulative ES products. These products are responsible for hemorrhages close to mechanical lesions, observed in infected gut mucosa [28, 40]. In the second scenario, the total crude extract (EC) of larvae was used to mimic senescent or dying larvae, because humans represent an accidental host for anisakids where infective larvae fail to fulfil the life-cycle and eventually die. After eliciting a potent eosinophilic inflammation in the digestive tract during migration, larvae subsequently die in four weeks, on average [4]. Therefore, EC represents an admixture of ES products and all other components of the parasite body.

Our results showed for the first time that both types of *Anisakis* products lead to increased production of oxygen reactive species (ROS) (Fig. 1c), which were more elevated when cells were exposed to EC products. Oxidative stress represents a misbalance in the production of free radicals and oxygen metabolites (or reactive oxygen species, ROS) compared to the rate of their elimination by antioxidants, which consequently leads toward damage of cell organelles and their biomolecules [41]. It is postulated that ROS have a role in many diseases among which chronic inflammation and cancer have a prominent significance [42]. Phagocytes in particular inactivate pathogens mostly through the action of cytotoxic compounds that they endogenously produce, as highly reactive oxygen and nitrogen species (RNS) [43, 44]. Individuals with an imbalance in ROS and RNS production are more susceptible to infection, having an increased rate of infection-induced mortality (reviewed by [45]). In addition, epidemiological and experimental studies have recognized that regardless of its cause (e.g. biological, chemical and/or physical), the association between the chronic inflammation with an increased risk of several human cancers is significant [46]. Up to 15–20% of worldwide malignancies have been epidemiologically attributed to infection [47], while many cancers have been associated with pathogens that induce strong inflammatory response of the host, in particular the release of ROS and RNS [48]. Cells become damaged by cytotoxic products excreted during chronic inflammation, and are consequently replaced by new cells, although their genomic integrity is continuously subjected to alteration induced by elevated ROS and RNS production [49]. Such oxidative stress is present in all of cancer stages; initiation, promotion, and progression [50–52], highlighting the epidemiological importance of induced ROS production in recurrent or chronic *Anisakis* infections.

We have provided evidence that a normal fibroblast cell line undergoes two different pathways, depending on the type of parasitic product used for treatment. Whilst ES products are water-soluble, EC fraction encompasses the extraction of different components present in cuticle of the parasite along other products that the larva normally excretes/secretes. Generally, in helminth infections, including those by *Anisakis*, pathological changes in the gastrointestinal tract result from the interaction of the larva and its products secreted during tissue penetration, and the complex immune reaction of the host. Such secreted products, which are mainly released from dorsal esophageal glands and excretory cells of the parasite, are strong proteolytic enzymes that induce mechanical tissue damage by degrading the extracellular matrix [53–58]. In the case of ES-treated cells, observed mortality of the fibroblast line is strongly dose/time-dependent, with corresponding exacerbation of oxidative stress (Fig. 1a, c). Such an environment determines the type of molecular markers expressed, which are directed towards stimulation of inflammatory pathways as mechanisms of cell defense, and apparently cell proliferation.

Observed toxicity and ROS production in treated cells induced an increase in p53 levels, demonstrated both by immunoblotting and RT-PCR (Fig. 1d and Additional file 2: Figure S2). Protein p53, defined as a “genome guardian”, plays an intracellular key role in the maintenance of genetic stability; it is a transcription factor with a central role in cell response to a variety of physical and chemical stressors, among which are agents responsible for DNA damage, oxidative stress and hypoxia [59]. Different stress signals are transduced mainly by p53 capacity to act as transcription factor and its regulation of expression of numerous genes that control cell-cycle progression, apoptosis, DNA repair and stress-response related functions [60]. An increase in p53 is associated with inflammation, where oxidative stress conditions are also present [42, 61]. However, it has been demonstrated that p53 has both a pro-oxidative [62] and anti-oxidative effect [63]. Those contrasting effects are reflected by p53-induced expression of pro- and anti-oxidant genes. Antioxidative functions of p53 are associated with the induction of target genes, among which is the glutathione peroxidasis-coding gene [64]. The latter decreases ROS levels during physiological, but not lethal stress. In contrast, pro-oxidative effects are consequence of induction of pro-apoptotic genes, defined as PIG (p53-induced genes), coding proteins whose activity increases intracellular ROS [62]. Therefore, an increase of ROS in *Anisakis*-treated cells could have been a result of intensive stress that consequently had p53-promoted induction of pro-oxidant proteins.

The treatments also induced TNF activation and, consequently, an increase of *c-jun* and *c-fos* expression (Fig. 1d and Additional file 1: Figure S1). Upregulation of these markers is associated with inflammatory onset, largely demonstrated during manifestation of the anisakid infection [4], and not the apoptosis, which would have been expected at first glance. The main difference between ES and EC was in strong upregulation of TNF, *c-jun* and *c-fos* in the former, and Hsp70 in the latter. Tumor necrosis factor TNF-α is implicated in a wide array of physiological functions, infections, autoimmune disorders and carcinogenesis, as a pleiotropic proinflammatory cytokine [65]. The interaction between TNF-α and its receptors Tumor Necrosis Factor Receptor 1 (TNFR1) and Tumor Necrosis Factor Receptor 2 (TNFR2) [66], starts a multiple intracellular signaling that activates among others, NF-κb pathway and specific mitogen-activated protein kinases (MAPK) as p38 and Jun N-terminal kinase (JNK) [67]. Cell response to such activation will depend on a fine interaction between apoptotic and anti-apoptotic mechanisms; if the TNFR1 intracellular domain called Death Domain (DD) specific molecular adaptors are relegated, signal transduction towards apoptosis will be activated. In contrast, when the TNF Receptor Associated Factor (TRAF) protein family is activated, it induces activation of transcription factors like NF-κb and JNK that consequently activate nuclear transcription complex AP1 (Activator Protein-1), composed of subunit c-jun and c-fos. These are factors important in normal cell survival, proliferation, cell cycle progression and inflammatory and immune response [68]. However, they are sensitive to changes in cell redox balance [69] and their transcriptional activities are particularly enhanced in uncontrolled tumor cell proliferation [70, 71]. Apparently, induction of redox-sensitive pathways during tumor cell proliferation is necessary, because the process itself demands increased energy requirements and consequent increased production of ROS metabolites might damage proliferating tumor cells [72]. This indicates that TNF induced by ES *Anisakis* products is not a prerequisite of apoptosis, but an early hallmark for consequent activation of kinases that supports inflammation and cell proliferation. This is congruent with [73], who observed that *A. simplex* larvae activate human eosinophils by phosphorylation of p38 MAPK and superoxide anion production, consequently increasing expression of surface receptors CD11b and CD69 on eosinophils and their degranulation. The authors suggested that p38 MAPK-mediated ROS production is required for the *Anisakis*-induced activation of eosinophils. However, we need to underline that, if c-jun (upregulated in ES-treated cells) undergoes phosphorylation at 63 and 73 serine site, instead of cell proliferation, c-jun induces anti-apoptotic protection [74]. As we are not able to discern between downstream fate of c-jun, we would refer to ES products to have cell proliferative and anti-apoptotic effect.

**Fig. 2:**
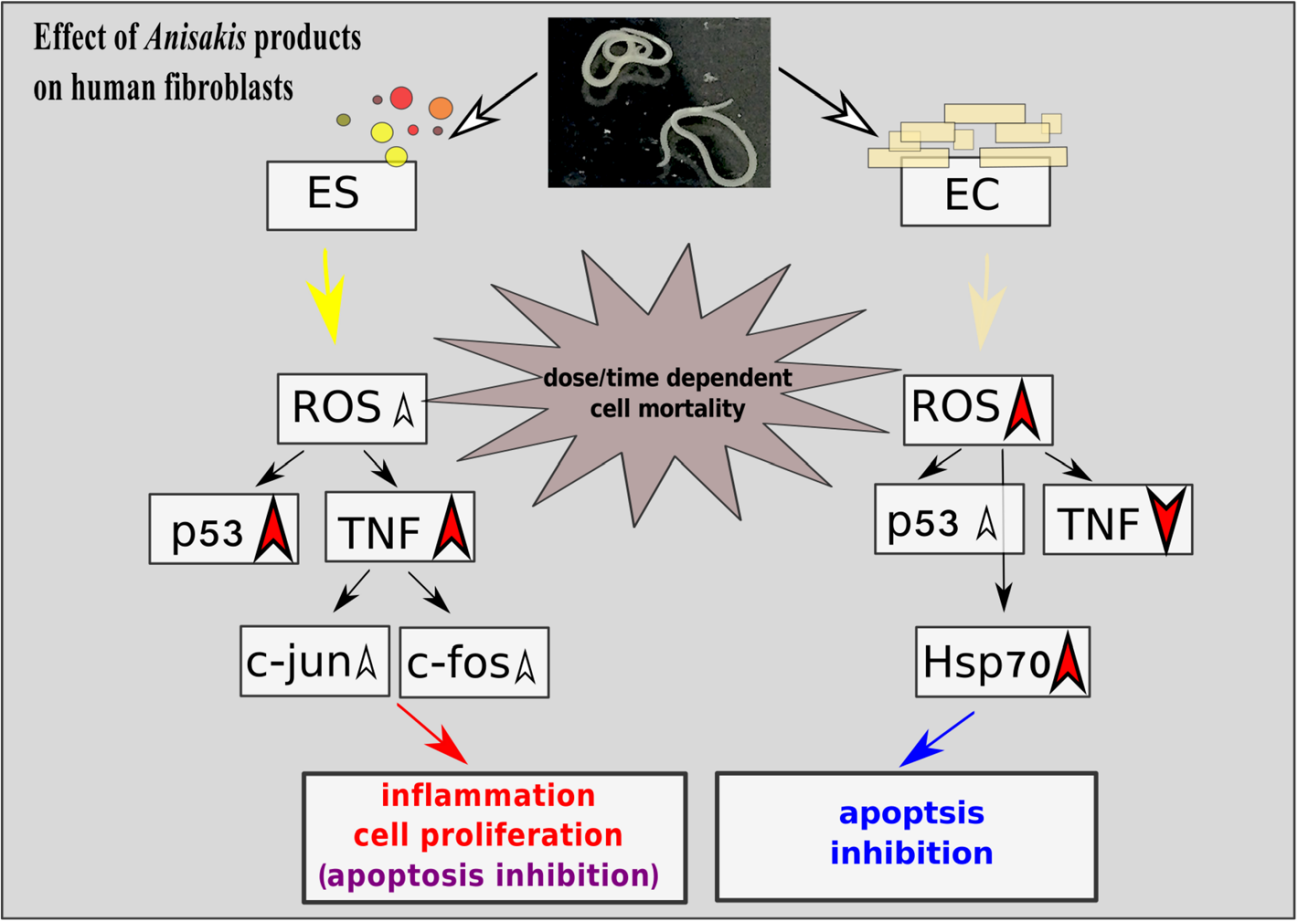
Putative pathways of normal fibroblast HS-68 cell lines response after exposure to *Anisakis* excretory/secretory (ES) and crude extract (EC) products. *Red arrowheads* turned up or down represent significantly upregulated or downregulated markers respectively, and *white smaller arrowheads* represent moderately upregulated proteins and genes

Although the same targets (c-jun and c-fos) are expressed in EC-treated cells, they show lower levels compared to ES-treated cells, while TNF is markedly downregulated. This suggests other signaling pathways involved in cell reaction to EC that do not result in cell proliferation but inhibition of apoptosis, as marked by strong upregulation of Hsp70 (Fig. 2). In lymphoma and myelogenous leukemia cells, suppression of TNF and another proinflammatory cytokine interleukin 1 (IL-1) were shown to downregulate the expression of active NF-κB and consequently inhibit tumor growth [75]. Additionally, a mild oxidative stress would lead to modest NF-κB activation, while extensive oxidative stress as observed in EC-treated cells could inhibit NF-κB [76], further supporting the inhibition of cell proliferation.

Therefore, while EC-treated cells do not show highly elevated markers for cell proliferation, they do show activation of Hsp70, supportive of the apoptosis inhibition. Its role in numerous conditions related to stress, including the oxidative stress, as well as its capacity to prevent cell death by apoptosis has been widely reported [77, 78]. During over-expression, Hsp70 relegates the proapoptotic Bax protein avoiding the formation of apoptosome, and the activation of caspases, which trigger apoptosis [78, 79]. This is in line with the composition of EC reported previously. Namely, two allergens - Ani s 4 and Ani s 6, both isolated from the parasite crud extract [80, 81], have been reported to act as inhibitors of cysteine protease [35, 37], a group also encompassing caspases, suggesting a role in blocking apoptotic signal transduction. Similarly, it was demonstrated that a rodent malaria parasites *Plasmodium yoelii* blocks apoptosis in the majority of infected hepatocytes that also resist Fas-mediated apoptosis (or tumor necrosis factor receptor superfamily member 6; TNFRSF6). In contrast, apoptosis was dramatically increased in hepatocytes infected with attenuated parasitic liver stages, suggesting that an anti-apoptotic host environment favors parasite survival [82]. There are cases where parasite-mediated induction of apoptosis helps larval stages to evade humane immune cells and establish the infection, as in the lung fluke *Paragonimus westermani* whose metacercarial excretory and secretary products induce caspase-3 in man eosinophils [83]. However, in the case of non-permissive hosts, host-mediated apoptosis induces apoptosis in the parasite, resulting in its successful elimination. This has been observed in the reed vole, *Microtus fortis* and *S. japonicum* system, the former recognised as the only mammalian host in which schistosomes fail to mature or cause significant pathology [84]. This suggests that interaction through apoptotic pathways of both the parasite and its host are likely dependent upon the tightness of their evolutionary history, sometimes promoting parasite infection, sometimes inhibiting it; the actual role of these complex pathways in *Anisakis* infection still remain to be studied.

In contrast to ES, EC-treatment demonstrated an increased concentration-dependent toxicity manifested as elevated cell mortality and ROS production. In such toxic conditions cells are directed towards the inhibition of apoptosis, and not towards inflammation or proliferation, which would represent potential alternatives or cells’ safeguard paths, as observed in ES-treatment (Fig. 2).

Finally, significant induction of p53 in fibroblasts treated by both product types was expected and supports the behavior of above discussed cell markers. Being a typical marker of cell response to a variety of stressors, activated at specific check points during cell transition through G1/S, G2/M and M phase of the cycle in case of DNA damage [85], it provides evidence of a significantly negative effect on the DNA induced by *Anisakis* products. In an extremely pro-oxidative condition (as in EC-treated cells), oncoprotein p53 would behave as a pro-oxidant, adding up to the elevated cell damage and mortality. In *Schistosoma haematobium*-induced bladder carcinoma, p53 has been connected to aggressive urothelial and squamous cell carcinomas, as well as in non-malignant parasite-infected tissues, suggesting its potential as a diagnostic marker for early detection of *Schistosoma*-infected patients at risk of developing of bladder cancer [86]. Similar approach could be useful in case of patients with incidence of chronic anisakiasis.

## Conclusions

Our observations are in line with the research of larval products biochemistry supporting that *Anisakis* sp. ES products contain pro-inflammatory molecules [87] that are able to diverge ES-treated cells towards inflammation, cell proliferation and inhibition of apoptosis. The presence of highly toxic substances contained in the nematode cuticle and body (EC) [88] instead, redirects fibroblasts towards the inhibition of apoptosis during a chronic infection.

## Additional files

Additional file 1: Figure S1. Regression analysis between cells vitality and ROS production at 48 h in control cells (CO) and after treatment with excretory/secretory (ES) and crude extract (EC) products (*n* = 9 for each treatment). (TIF 179 kb)
Additional file 2: Figure S2. Western blotting of (a) p53, (b) hsp70 and (c) TNF proteins detected in normal fibroblast HS-68 cell lines after 48 h exposure to a 0.1% concentration of *Anisakis* excretory/secretory (ES) and crude extract (EC) products: Lane 1: control; Lane 2: ES exposure; Lane 3: EC exposure, Std, mix of standard proteins as molecular markers. **P* < 0.05, ***P* < 0.0001 in respect to the control. Images are representative of at least three separate experiments. p53 (ANOVA *F*_(2,6)_ = 43.9, *P* < 0.05); hsp 70 (ANOVA *F*_(2,6)_ = 269.4, *P* < 0.0001); TNF (ANOVA *F*_(2,6)_ = 20.6, *P* < 0.05) *Abbreviation*: FI, fold increase respect to the control level. (DOCX 79 kb)

## Abbreviations

AP1: Activator protein-1
CD11b: Cluster of differentiation 11b (integrin alpha M chain)
CD69: Cluster of differentiation 69
c-fos: Finkel-Biskis-Jinkins murine osteosarcoma viral oncogene homolog
c-jun: JUN proto-oncogene
CO: Control treatment
DCF-DA: Dichlorodihydrofluorescein-diacetate
DD: Death Domain
EC: Crude extract
ES: Excretory/Secretory products
*HRAS*: *Harvey rat sarcoma* viral oncogene homolog
HS-68: Human normal fibroblast cell line
HSP70: Heat-shock protein 70
IL-4: Interleukin 4
JNK: Jun N-terminal kinase
*KRAS*: *Kirsten rat sarcoma* viral oncogene homolog
MAPK: Mitogen-activated protein kinases
MTT: 3-(4,5-Dimethylthiazol-2-yl)-2,5-Diphenyltetrazolium Bromide
NF-κb: Nuclear factor kappa-light-chain-enhancer of activated B cells
NOS: Nitric oxide synthase
*NRAS*: *Neuroblastoma RAS* viral (v-ras) oncogene homolog
p38: Tumor protein 38
p53: Tumor protein 53
PIG: p53 induced genes
RNS: Reactive nitrogen species
ROS: Reactive oxygen species
Th2: Type 2 helper T cells
TNFR1: Tumor necrosis factor receptor 1
TNFR2: Tumor necrosis factor receptor 2
TNFRSF6: Tumor necrosis factor receptor superfamily member 6
TNF-α: Tumor necrosis factor α
TRAF: TNF receptor associated factor
