# Supplementary figures and images for: Anisakis pegreffii (Nematoda: Anisakidae) products modulate oxidative stress and apoptosis-related biomarkers in human cell lines
Source: Parasit Vectors. 2016 Nov 25;9:607. doi: 10.1186/s13071-016-1895-5 (PMC5124272; doi:10.1186/s13071-016-1895-5)

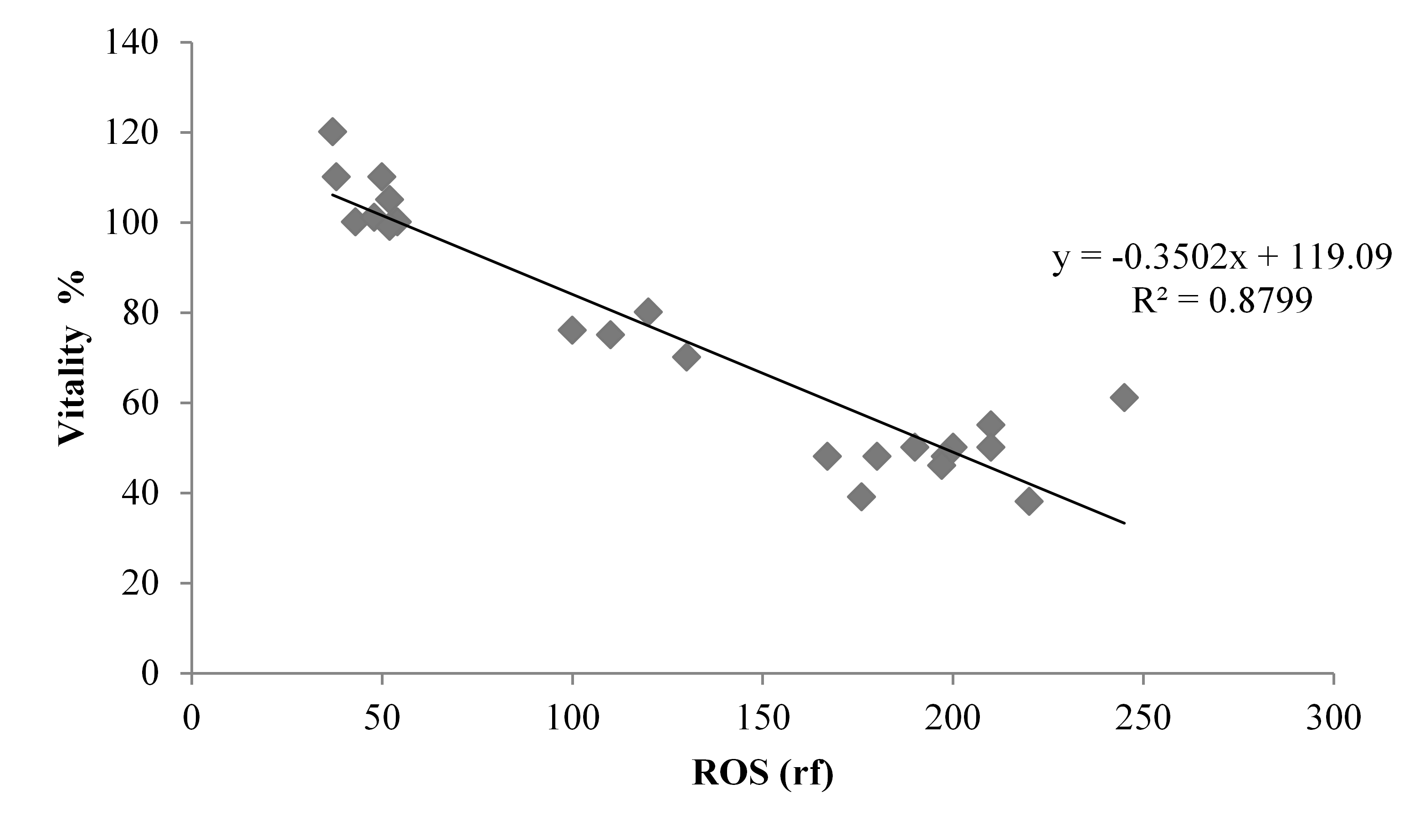

Supplement: Additional file 1: Figure S1. — Regression analysis between cells vitality and ROS production at 48 h in control cells (CO) and after treatment with excretory/secretory (ES) and crude extract (EC) products (n = 9 for each treatment). (TIF 179 kb) [file 13071_2016_1895_MOESM1_ESM.tif]

| **A** | **STD 1 2 3** |
| --- | --- |
| **-50kDa** | 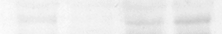 |
| ***FI*** | ***STD 1+ 0.1 1.45+ 0.1* 1.7+ 0.2**** |
| **B** | 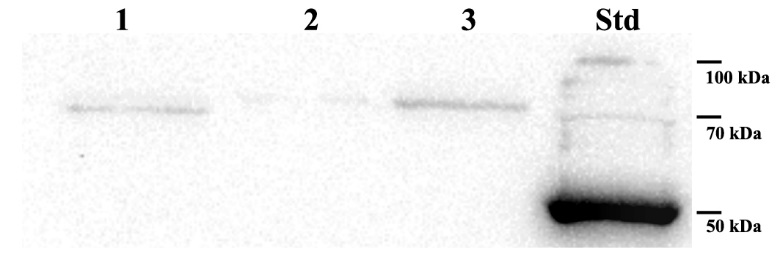 |
| ***FI*** | ***1+ 0.1 0.45+ 0.1* 1.7+ 0.2** STD*** |
| **C** | 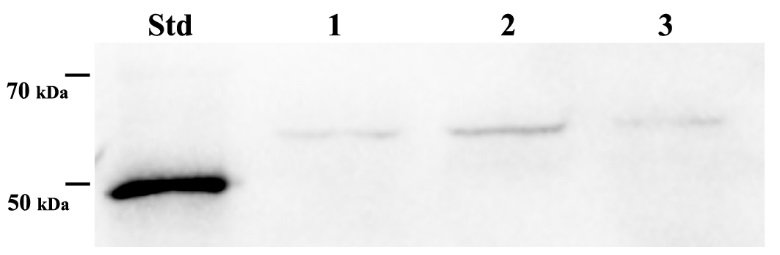 |
| ***FI*** | ***STD 1+ 0.1 1.85+ 0.13* 1.3 + 0.2*** |

Supplement: Additional file 2: Figure S2. — Western blotting of (a) p53, (b) hsp70 and (c) TNF proteins detected in normal fibroblast HS-68 cell lines after 48 h exposure to a 0.1% concentration of Anisakis excretory/secretory (ES) and crude extract (EC) products: Lane 1: control; Lane 2: ES exposure; Lane 3: EC exposure, Std, mix of standard proteins as molecular markers. *P < 0.05, **P < 0.0001 in respect to the control. Images are representative of at least three separate experiments. p53 (ANOVA F (2,6) = 43.9, P < 0.05); hsp 70 (ANOVA F (2,6) = 269.4, P < 0.0001); TNF (ANOVA F (2,6) = 20.6, P < 0.05) Abbreviation: FI, fold increase respect to the control level. (DOCX 79 kb) [file 13071_2016_1895_MOESM2_ESM.docx]
